# Multiscale analysis of a regenerative therapy for treatment of volumetric muscle loss injury

**DOI:** 10.1038/s41420-018-0027-8

**Published:** 2018-02-20

**Authors:** Carlos A. Aguilar, Sarah M. Greising, Alain Watts, Stephen M. Goldman, Chelsea Peragallo, Christina Zook, Jacqueline Larouche, Benjamin T. Corona

**Affiliations:** 1 Department of Biomedical Engineering, University of Michigan, Ann Arbor, MI USA; 2 Extremity Trauma and Regenerative Medicine, United States Army Institute of Surgical Research, Fort Sam Houston, San Antonio, TX USA; 3 Massachusetts Institute of Technology - Lincoln Laboratory, Lexington, MA USA

## Abstract

Skeletal muscle possesses a remarkable capacity to regenerate when injured, but when confronted with major traumatic injury resulting in volumetric muscle loss (VML), the regenerative process consistently fails. The loss of muscle tissue and function from VML injury has prompted development of a suite of therapeutic approaches but these strategies have proceeded without a comprehensive understanding of the molecular landscape that drives the injury response. Herein, we administered a VML injury in an established rodent model and monitored the evolution of the healing phenomenology over multiple time points using muscle function testing, histology, and expression profiling by RNA sequencing. The injury response was then compared to a regenerative medicine treatment using orthotopic transplantation of autologous minced muscle grafts (~1 mm^3^ tissue fragments). A chronic inflammatory and fibrotic response was observed at all time points following VML. These results suggest that the pathological response to VML injury during the acute stage of the healing response overwhelms endogenous and therapeutic regenerative processes. Overall, the data presented delineate key molecular characteristics of the pathobiological response to VML injury that are critical effectors of effective regenerative treatment paradigms.

## Introduction

Skeletal muscle comprises over 40% of body mass in lean individuals, is primarily responsible for coordinating voluntary movements and can readily adapt to its environment. After injury, skeletal muscle is capable of repairing and regenerating through a pool of resident stem cells (satellite cells) that activate, proliferate, differentiate, and fuse to form new or repair existing multinucleated myofibers^1^. However, after volumetric muscle loss (VML) injury^2^, which is a type of severe trauma that ablates resident cells and structures primarily responsible for regeneration, the intrinsic muscle regenerative process fails^3^. Instead, gross compartmental tissue fibrosis manifests^4,5^ and chronic functional deficits result. VML is a common clinical outcome after open or closed (e.g., crush injury requiring fasciotomy and tissue evacuation) extremity trauma, for which there are currently no regenerative standards of care.

The magnitude and location of VML injuries result in considerable heterogeneity and necessitate a cadre of therapies. For instance, a small VML defect isolated to a peripheral portion of a muscle unit may be best compensated by synergist hypertrophy mediated through physical therapy. Whereas, massive VML defects have been shown to not respond to conventional physical therapy and may require regenerative medicine augmentation. Regenerative treatments for VML have utilized various strategies^6–14^ but require significant further advancement to be of therapeutic benefit to patients presenting acute or chronic VML injury.

The fairly extensive number of regenerative therapies that have been tested for VML repair have proceeded to date without a comprehensive understanding of the pathophysiology of the injury response. Animal models of VML injury have described^15,16^ a dysregulated immune response that coincides with aberrant or muted muscle fiber regeneration and fibrosis, although the temporal coordination of these events and drivers of this response trajectory have not been elucidated. Without determination of a clearly effective therapy and lack of knowledge of the molecular phenomenology-mediating injury repair, further advancement of the field depends on understanding and mitigation of the host pathobiological response. Thus, a key step to advance translational medicine in this domain is to establish a comprehensive reference map of the various molecular patterns that drive the diverse sub-processes through time and identify critical factors that influence and modulate gene expression amplitude and dynamics. The formulated unbiased views of the pathophysiology can then serve as a guide to assess and enhance new therapeutic modalities for VML.

**Fig. 1:**
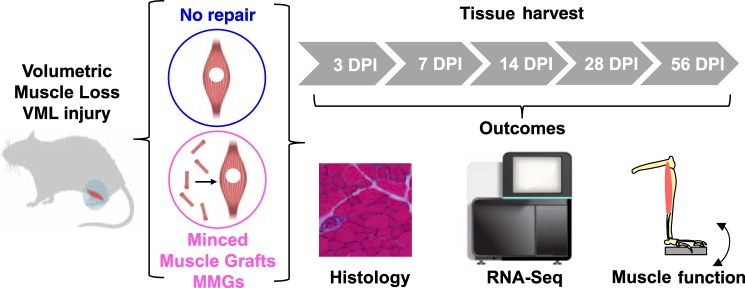
Schematic of experimental approach to define the molecular response to volumetric muscle loss (VML) injury and how transplantation of autologous minced muscle graft (MMGs) impacts regenerative trajectory. Injured and uninjured muscles were extracted at multiple days post injury (DPI) and characterized using histology, high-throughput sequencing of RNA (RNA-Seq), and muscle function

Herein, we administer a VML injury to a rodent model and track the molecular phenomenology and etilogy through multiple time points using muscle function testing, histology, and expression profiling by RNA sequencing (RNA-Seq) (Fig. 1). Next, we evaluate the effect of a regenerative therapy (orthotopic transplantation of autologous minced muscle grafts (MMGs)) and compare readouts of tissue and molecular function. Employing this approach, we were able to quantitatively understand the time course of tissue-level changes induced by a regenerative therapy and establish metrics for assessing progression through discrete healing state transitions. This schema provides a resource to understand the physiologic response to VML as well as to enable sensitive evaluation of regenerative therapies after severe trauma.

## Results

### VML induces fibrotic scarring and poor muscle regeneration

**Fig. 2:**
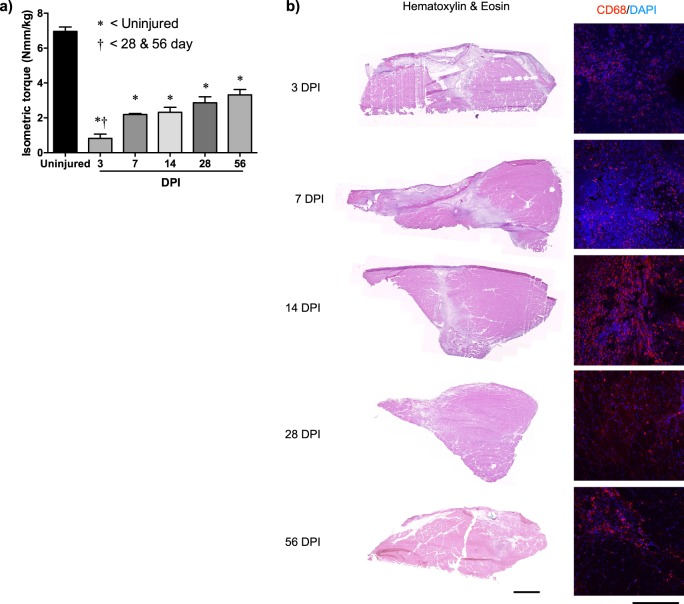
VML injury in the rat TA muscle induces chronic strength deficits and prolonged tissue damage. **a** TA muscle maximal isometric torque was elicited using common peroneal nerve stimulation in uninjured and VML injured (non-repair) muscle at the specified days post injury (DPI). Values are mean ± sem. *, †: *p* < 0.05. **b** Representative TA muscle hematoxylin and eosin (H&E) and macrophage (CD68) probed sections are presented. Scale bars for H&E and CD68 images are 1000 and 150 µm, respectively. TA tibialis anterior, VML volumetric muscle loss

VML injury was surgically created unilaterally in the rat tibialis anterior (TA) muscle and assessed for in vivo neural-evoked strength deficits intermittently post injury (Fig. 2a). At 3, 7, 14, 28, and 56 days post injury, ~88, 69, 67, 59, and 52% of strength was lost, respectively. Histologically, VML injury resulted in a clear muscle defect devoid of muscle fibers, and an associated influx of inflammatory cells over the ensuing weeks (Fig. 2b). Macrophages (CD68^+^ cells) persisted in the defect for weeks to months after injury (Fig. 2b). Consistent with previous studies demonstrating gross fibrosis within the VML wound bed^3,16,17^, evidence of muscle fiber regeneration within the defect was limited out to 56 days post injury.

### Temporal transcriptomes of VML-injured tissues display pathways associated with heightened and sustained inflammation in combination with excessive fibrogenesis

**Fig. 3:**
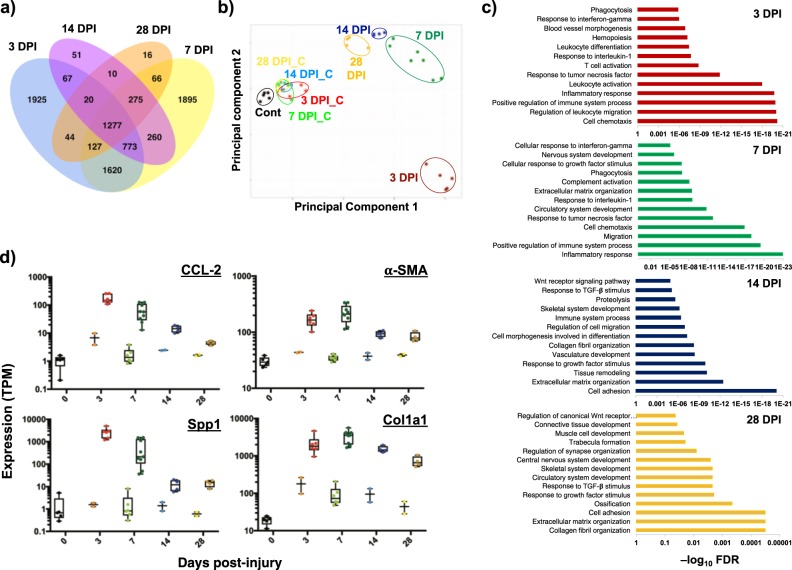
Characterization of molecular response to VML injury. **a** Venn diagram showing number of unique and overlapping differentially expressed genes for muscle tissues administered VML injury and extracted at different days post injury (DPI). Days 3 and 7 show the most unique differentially expressed genes. **b** Principal component analysis (PCA) of RNA-Seq data sets from control (cont), injured (example: 3 DPI), and uninjured (example: 3 DPI_C) data sets from different time points harvested show distinct separation between injured and uninjured samples. **c** Enriched pathways associated with differentially expressed genes from each time point. Enrichment scores are plotted as −log_10_(FDR), where FDR is the false discovery rate. **d** Individual graphs of gene expression plotted in TPM (transcripts per million reads) where uninjured samples are plotted first and injured samples are plotted second for each time point. CCL2: C-C Motif chemokine ligand 2, SPP1: secreted phosphoprotein 1, α-SMA: alpha smooth muscle actin, Col1a1: collagen type I alpha chain. VML volumetric muscle loss

To measure the molecular mechanisms contributing to poor muscle regeneration induced by VML, we performed expression profiling by RNA-Seq for unoperated controls, VML-affected muscles and unaffected contralateral muscle for multiple time points in the acute response phase (3, 7, 14, 28 days post injury). Biological and technical replicates for all three sample types demonstrated excellent correlations and reproducibility across all time points. Samples that did not meet adequate thresholds were discarded. In total, 16,020 mRNAs were detected (TPM > 1) for at least one time point; and 4,897 genes were observed to be dynamic (fold change > 2) at one or more time points (Fig. 3a). Principal component analysis (PCA) of the data sets revealed the contralateral uninjured tissues clustered together for all time points and in close proximity to control tissues (Fig. 3b). PCA also revealed the tissues extracted 3 days post injury displayed significant separation from other time points and the tissues progressed back toward the contralateral uninjured tissues as time progressed. However, for tissues extracted at 28 days post injury, a distinct separation was observed between the injured and uninjured tissues, indicative the tissue did not fully heal as measured by tissue transcriptome.

Pathway analysis of the differentially expressed genes for 3 days post injury revealed a large number of gene sets associated with chemotaxis, inflammation, and immune cell infiltration. Previous muscle regenerative studies^18,19^ have shown inflammation -related programs subsided after several days, but many of these genes remained upregulated over the entire time course (Fig. 3c–d). Detection of these transcripts was also consistent with immuno-staining of the injured muscle, whereby invasion of CD68^+^ cells (monocytes and macrophages) into the injured site was observed 14 and 28 days post injury (Fig. 2b). At 7 days post injury, many of the pathways associated with the inflammatory response and immune system remained upregulated, such as the Complement pathway. Previously, sustained activation of the Complement system in regenerating muscle was shown to stimulate Wnt signaling^20^ and promote fibrosis by attenuating satellite cell proliferation^21,22^ and increasing fibroblast production of collagen^23^. Consistent with this observation, strong increases in expression of a family of genes-associated connective tissue cells (Col1a1, α-SMA/Acta2, S100a4) along with extracellular matrix (ECM) deposition and remodeling such as structural components (collagen and elastin) were detected. This result was also in agreement with histological observations of fibrotic supplantation of muscle tissue (Fig. 2b) at this time period. At 14 and 28 days post injury, fibrogenic pathways such as Wnt, transforming growth factor beta (TGF-β), and other ECM pathways remained enriched and indicated that the regenerative process began to fail and been replaced by fibrosis. Taken together, these results suggest VML induces strong and persistent overexpression of inflammatory transcripts, which in turn provokes dysregulated ECM deposition and may inhibit satellite cell repair.

### Orthotopic transplantation of autologous MMGs stimulates muscle repair and regeneration

**Fig. 4:**
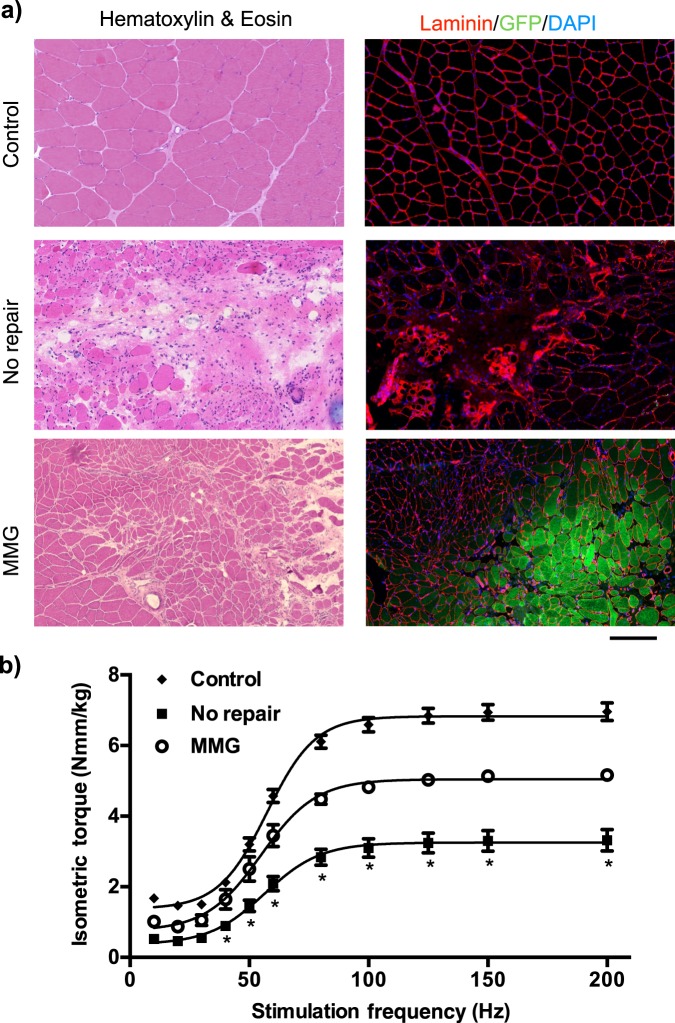
Autologous MMGs promote partial muscle fiber regeneration and functional recovery. **a** TA muscle sections stained with hematoxylin and eosin or probed for laminin, GFP, nuclei from control (uninjured), VML injured no repair and minced graft repaired muscles 56 days post injury are presented. Scale bar is 200 µm. **b** Maximal TA muscle isometric torque was measured as a function of stimulation frequency in each group at 56 days post injury. Values are mean ± sem. *p < 0.05. MMG minced muscle graft, TA tibialis anterior

Autologous MMGs have previously been shown to orchestrate de novo muscle fiber regeneration^11,24^. MMGs derived from syngeneic green fluorescent protein (GFP) Lewis rats were surgically implanted in a collagen gel in the VML defect site and progenitor cells derived from the minced grafts were observed to contribute to de novo muscle fiber regeneration, evidenced by the presence of GFP^+^ fibers 56 days post injury (Fig. 4a). Compared to non-repaired muscles, minced graft repair of VML injury significantly improved TA muscle strength across a range of peroneal nerve stimulation frequencies by 56–95% by 56 days post injury; however, minced graft repaired muscles still presented an ~26% deficit of maximal strength (Fig. 4b).

### Gene expression programs after regenerative treatment display similar response trajectories to non-repaired tissues

**Fig. 5:**
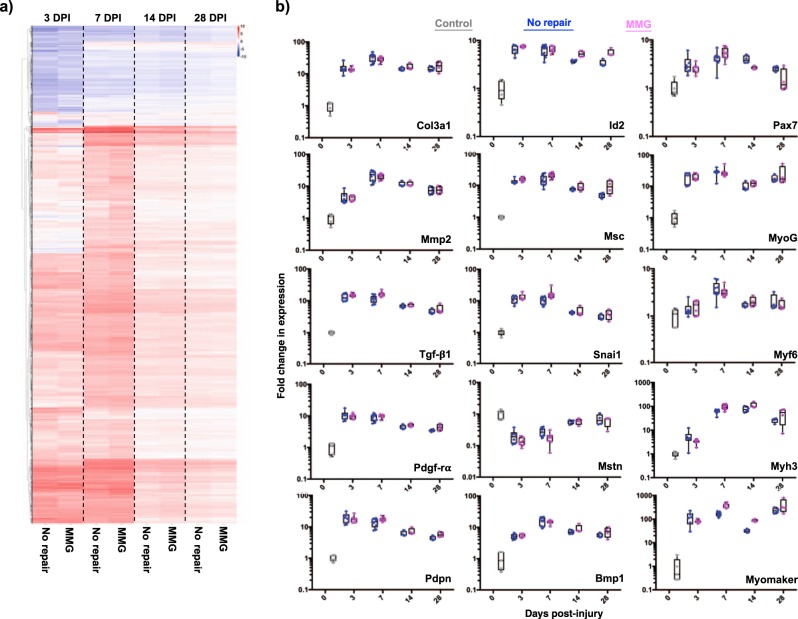
Transcriptional response of regenerative (MMGs) treatment for VML show marginal effects compared to non-repaired tissue at time points sampled. **a** Heatmap of differentially expressed genes (injured vs. uninjured) for each treatment and time point. Data are plotted as union of biological replicates. **b** Individual box plots of gene expression plotted as fold change (injured vs. uninjured) for four different time points (Sham-gray, No Repair-blue, MMG-purple). DPI days post-injury, MMG minced muscle graft, VML volumetric muscle loss

To quantitatively understand how a regenerative medicine therapy converges to positively influence endogenous gene expression programs after VML, whole-tissue RNA-Seq was performed for each time point and condition. Differential expression (DE) analysis and hierarchical clustering of the data sets through time revealed highly similar patterns for the non-repaired samples with those that were treated with MMGs (Fig. 5a). This observation is clearly illustrated by a family of genes nominally associated with fibrosis (collagen 3-Col3a1, matrix-metalloproteinase 2-MMP2, TGF-β1, platelet-derived growth factor receptor α-PDGFRα, podoplanin-PDPN), where the expression pattern appeared invariant with treatment (Fig. 5b). This result was also viewed for multiple genes associated with negative regulation of myogenesis (inhibitor of differentiation 2-Id2, musculin-MSC, snail family transcriptional repressor 1-Snai1, myostatin-MSTN, bone morphogenetic protein 1-Bmp1). Comparing the genes associated with positive myogenic regeneration (paired box protein 7-Pax7, myogenin-MyoG, myogenic factor 6-Myf6, embryonic myosin heavy chain-Myh3, Tmem8c-Myomaker) from the unrepaired samples with those treated with MMGs, increases in expression were observed over the time course for both types of samples but did not vary considerably with treatment. This result suggests that the acute VML response results in a robust tissue transcriptome characterized by a fibrotic pathology that suppresses relative expression of canonical myogenic pathways, regardless of treatment.

## Discussion

Currently, very little is known about the global molecular response after VML and why this injury exceeds skeletal muscle’s endogenous regenerative capabilities through satellite cells. Recently, we administered a VML injury to a swine model^16^ and observed upregulation of inflammatory and fibrotic transcriptional programs at 3 months post injury. Understanding the molecular signals that induce the response of skeletal muscle to adopt a non-regenerative phenotype and when these signals are presented is paramount in advancing our understanding of VML pathogenesis and therapeutic efficacy. In this study, we performed multi-dimensional profiling of VML-injured tissues during the healing process using muscle function, histology, and transcriptomics and compared the response to treatment with autologous MMGs. Profiling of unfractionated muscle tissue from the VML injured site enabled detection of transcripts from multiple cell types and insights into tissue-level responses.

For muscles that were non-repaired after VML, in all time points the tissue appeared to be overwhelmed by persistent inflammatory and fibrogenic signals. Histological observation of the non-repaired muscles highlighted inadequate muscle fiber regeneration and a dense population of macrophages in the defect area. This result is consistent with sustained upregulation of multiple components of the Complement system, which has previously been shown to impair muscle regeneration^20^ and promote fibrotic deposition. In contrast to muscle regenerative studies, whereby the cellular infiltrate transitions^25^ from a pro-inflammatory population to a primarily anti-inflammatory phenotype after several days, many of the inflammatory and immune-related signaling pathways remained upregulated over the entire time course. Persistent inflammation and inadequate debris clearance during muscle regeneration have been shown to influence fibrosis by inducing fibro-adipogenic progenitors (FAPs)^26,27^ to proliferate and differentiate into fibroblasts or adipocytes. We viewed sustained transcriptional overexpression of PDGFRα, a marker for FAPs, in parallel to sustained increases in expression of TGF-β1. Increases in TGF-β1 signaling have been shown to block FAP apoptosis and induce fibrogenic differentiation^28^, which our current findings suggest. Moreover, elevated TGF-β1 has been shown to suppress the regenerative functions of satellite cells^29^. The collective integration of these gene expression programs for non-repaired tissues suggests a model of continuous infiltration of immune cells and perpetual inflammation that in turn regulates mesenchymal progenitors and cells to produce excess matrix^30^. A similar model of chronic inflammation that contains both pro-inflammatory and anti-inflammatory immune cells has been shown for Duchenne muscular dystrophy, which also uniquely exhibits fibrosis and FAP infiltration and differentiation^31^.

The similar gene expression programs generated from VML-injured tissues without and treated with MMGs suggests unrelenting immune and fibrotic responses following VML injury. These results support the hypothesis that persistent inflammation and strong fibrosis overwhelm and dampen myogenic repair. Previously^11,32,33^, MMGs were found to promote muscle regeneration after extended post-surgical times (8–16 weeks), but in the early time periods sampled here (3–28 days) myogenic regenerative expression patterns did not vary considerably compared to non-repaired samples. These early time periods were deterministically sampled, as immediate interventions have been shown to favorably promote long-term outcomes for VML. However, multiple factors may be contributing to prevention of regenerative pathways from these treatments during the early time periods sampled such as the strong upregulation of negative regulators of myogenic differentiation such as TGF-β, SMAD2/3, Snai, musculin (MSC), myostatin (MSTN), and inhibitor of differentiation (Id) proteins (Id2 and Id3). Since muscle repair and regeneration canonically utilizes the sequential activation of specific basic helix-loop-helix transcription factors^34^ (MyoD, MyoG, and Mef2), potent overexpression of negative regulators may block these factors from appropriately activating myogenic genes. Thus, even if satellite cells or differentiated progenitors migrate or are transplanted into the defect site, their differentiation is initially obstructed by overexpression of negative regulators.

Another factor that may be contributing to suboptimal regenerative outcomes and inhibiting treatment efficacy at the time periods sampled is the aberrant deposition and remodeling of ECM in the defect area. Multiple ECM transcripts were strongly upregulated after VML such as fibrillar collagens, lysyl oxidase, and proteoglycans in comparison to proteases that breakdown matrix. The resulting dense composition of the deposited ECM may confer differential integrin signaling^35^ for satellite cells or myogenic progenitors in the defect site, which in turn would augment their differentiation and fusion. Loss of adhesive cues and increases in integrins^36^ (which were also detected) has previously been shown to alter muscle stem cell dynamics in aged tissues^37^ and produce weakened healing responses. Thus, the coupled action of negative regulators and dysregulated ECM produce an environment that is unfavorable for resident or transplanted myogenic cells to mediate optimal myogenic outcomes.

**Fig. 6:**
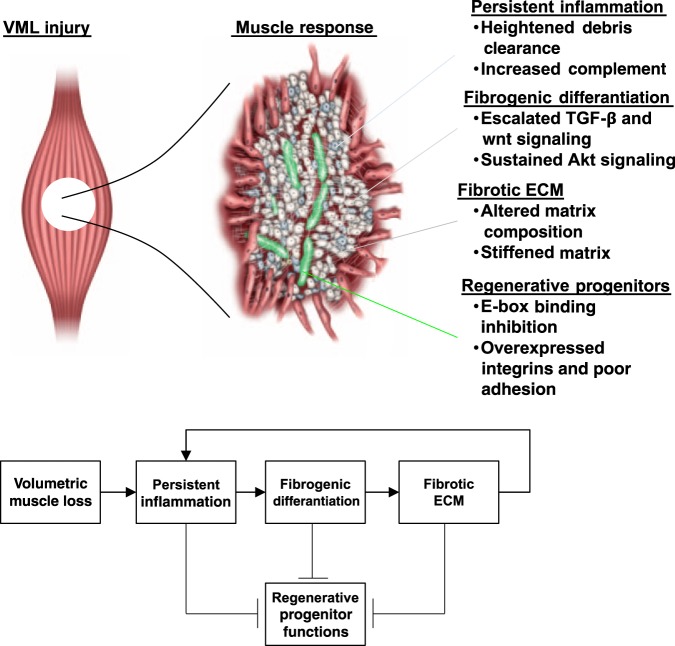
VML induces formation of an inhibitive feedback response loop that modulates the regenerative actions of myogenic progenitors. The abrupt tissue removal engenders a sustained inflammatory response that invokes fibrogenic cells to differentiate and produce excess matrix. The modified matrix and milieu in turn modifies the regenerative functions of myogenic progenitors and prevents healing. ecm extracellular matrix, VML

Integrating these results implies that VML injury drives formation of an inhibitive feedback loop^38^ that effectively limits therapies such as autologous grafts during these early periods (Fig. 6). Thus, in order to treat the response that results from a severe VML insult, treatment strategies need to be directed toward amelioration of this feedback loop. Since the immune response is the first reaction after VML injury, modulating early inflammatory effects may be effective for subsequent treatments (such as with progenitors). Indeed, recent approaches have taken aim at modulating early immune-related responses in the remaining musculature after VML injury to improve muscle regeneration^10,39^. Previously, it was shown that the behavior of mesenchymal progenitors^40^ and their differentiation into matrix-producing cells was affected by pro-inflammatory and sustained TGF-β signaling. Thus, immuno-modulatory drugs and technologies may be beneficial therapies but require consideration of their influence on the FAP population and subsequent differentiation. Another avenue of therapies requiring further investigation that may play an effective role when combined with immuno-modulation are anti-fibrotic agents^41^, which have been shown to limit pro-fibrotic TGF-β effects and persistent protein kinase B (Akt) signaling that perpetuate the feedback loop. Anti-fibrotic agents can induce apoptosis of FAPs and differentiated fibrogenic cells and when combined with inhibition of pro-inflammatory signals may permit a more favorable environment for myogenic regenerative cells to “break” the feedback loop. Combinatory treatment of inflammatory and fibrotic signals has previously been shown to attenuate muscular dystrophy pathologies^42,43^ and requires further investigation for treatment of VML. The generation of a more favorable microenvironment would then provide invading or transplanted myogenic progenitors to properly differentiate and regenerate tissue. In addition to encountering a more favorable environment, it was shown myogenic progenitors in fast twitch muscles protect against fibrosis via emission of exosomes containing microRNA-206 (miR-206)^44^, which represses ribosome-binding protein 1, a regulator of collagen biosynthesis. Thus, restoring the lack of or reduced capacity of satellite cells in the defect area may simultaneously attenuate fibrosis^45^ and promote regeneration.

In summary, our work provides definitive evidence of the molecular networks that underpin the VML response and progressive degeneration and fibrosis. We used a large-scale data approach to understand how these networks are adjusted when treated with a regenerative treatment strategy. The results of a common response behavior for both treated and unrepaired samples resemble muscular dystrophies, non-dystrophic myopathies, and amyotrophic lateral sclerosis, where continuous cycles of myofiber degeneration and incessant inflammation drive fibrosis. Accordingly, the mechanisms that ultimately drive impaired tissue regeneration after VML suggest this injury may be effectively treated with therapies for dystrophic muscle or other chronic pathologies.

## Materials and methods

### Animals

A total of 76 Lewis rats were used in this study. All animal procedures were approved by the Institutional Animal Care and Use Committee and were conducted in compliance with the Animal Welfare Act and in accordance with the principles of the Guide for the Care and Use of Laboratory Animals at the United States Institute of Surgical Research. Inbred male Lewis rats (350–400 g; ~4 months of age) were purchased from Harlan Laboratories (Envigo) and a subset of Lewis GFP rats (*n* = 4) were purchased from the Rat Resource Center, all rats were housed in a specific pathogen-free animal facility and given food and water ad libitium. All rats received a pre-surgical (~30 min prior) administration of buprenorphine-SR (1.2 mg/kg; s.c.) for pain management. No adverse events were observed.

### VML injury and repair

Rats underwent surgical creation of a VML injury in the left TA muscle using aseptic technique. Briefly, the lateral incision was made along the lower leg and the skin and fascia overlying the TA muscle were reflected. A spatula tool was placed under the middle third of the TA muscle and a 6-mm biopsy punch was used partially ablate muscle tissue from the region of the muscle. Approximately, 1-mm width of tissue remained on either side of the defect, which provided continuity of proximal and distal regions of the remaining muscle mass. The wet weight of the excised tissue was measured (~86 mg). A subset of rats underwent VML injury repair with autologous MMGs (~1 mm^3^ pieces of muscle tissue). The minced grafts were prepared from the excised tissue using Vanna scissors. The fascia was closed using 6-0 Vicryl and the skin was closed using surgical staples.

### Muscle histology

TA muscle tissue isolation and freezing were performed as described previously^11^. The muscle was frozen in melting isopentane and stored at −80 °C until further analysis. Sections of 8-μm thickness were stained with hematoxylin and eosin (H&E). Composite brightfield images of the complete TA muscle were acquired using Axio Scan.Z1 microscope and ZEN imaging software (Carl Zeiss Microscopy). Additional labeling of muscle sections was completed by combinations of antibodies and probes including DAPI (1 µg/ml; Molecular Probes D21490), monoclonal anti-mouse CD68 (1 µg/ml; Biorad MCA341R), monoclonal anti-rat laminin (2.5 µg/ml; Abcam ab11576), and polyclonal anti-goat GFP (1 µg/ml; Abcam ab6673). Appropriate isotype-specific-conjugated secondary antibodies (1:200; Molecular Probes A21125, A11055, or A21202) were then applied. Fluorescent imaging of the TA muscle was conducted with an Olympus FluoView FV1000 laser scanning confocal microscope (Olympus America Inc., Melville, NY) mounted on an inverted Olympus IX81 microscope and equipped with Multi-line Argon (458, 488, and 515 nm), HeNeG (543 nm), diode (405 nm), and diode (635 nm) lasers using an Olympus UPLSAPO 20×/0.85 N.A. oil immersion lens. All images were acquired as 12-bit multi-TIFF files.

### Muscle functional assessment

In vivo functional testing of TA muscles was performed using methodology previously described^11^. Briefly, TA muscle in vivo physiological properties were measured in anesthetized rats (isoflurane 1.5–2.0%) using a dual-mode muscle lever system (Aurora Scientific, Inc., Mod. 305b). Subcutaneous needle electrodes were inserted in the posterior compartment of the lower limb on each side of the common peroneal nerve. Optimal voltage (2–5 V) was set with a series of tetanic contractions (5–10 contractions; 150 Hz, 0.1 ms pulse width, 400 ms train). Then, a skin incision was made at the antero-lateral aspect of the ankle and the distal tendons of the extensor digitorum longus and extensor hallicus longus muscles was isolated and severed above the retinaculum^11^. TA muscle maximal isometric tetanic torque was measured as a function of stimulation frequency (10–200 Hz) with the ankle at a right angle. The servomotor input and force and displacement transducer outputs are controlled and acquired, respectively, using a PC equipped with a data acquisition board (National Instruments) and custom designed Lab View (National Instruments)-based software program.

### RNA extraction, quantification, preparation, and sequencing

TA muscles were harvested from euthanized rats, immersed in Trizol (Thermo Fisher) and snap frozen on liquid nitrogen and stored at −80 °C for subsequent use. The tissues were then thawed, homogenized (Tissue Ruptor, Qiagen) for 30 s at room temperature and total RNA was isolated from the slurry using the miRNeasy Mini Kit (Qiagen) as per the manufacturer’s instructions. RNA concentration and integrity were measured with a Nanodrop spectrophotometer (Nanodrop 2000c) and Bioanalyzer (Agilent 2100), respectively. Samples that did not pass quality metrics (RIN > 7) were precluded from further processing. 500 ng of isolated total RNA was used to produce cDNA libraries using the Truseq protocol (Illumina), as per the manufacturer’s instructions. Individual libraries were pooled and sequenced using 76 base-pair paired-end reads on a Hi-Seq 2500 in high output mode to an average depth of 35 M reads per library.

### RNA-Seq data processing

High-throughput RNA sequencing reads were pseudo-aligned to the Genbank-annotated *Rattus norvegicus* (Rat) genome (build 6.0), transcript abundances were quantified and gene counts were generated using Kallisto^46^ with default parameters. To determine sample quality for downstream analysis, per sample read depth was measured, and outliers were detected by conducting PCA on the log_2_-transformed gene counts. Samples that contained poor read depth (less than 5 million reads) or were determined to be outliers by PCA were removed from downstream analysis.

DE of gene counts was calculated using the R/Bioconductor implementation of DESeq2^47^. Analysis of expression data, such as set analysis based on treatment-specific expression profiles over time, PCA of treatment by time gene profiles, and correlation of inter-treatment gene expression were performed with the R base package. DE of genes in treated samples relative to uninjured controls was determined using a log_2_ fold-change threshold greater than 1 or less than −1 and significance was measured using an adjusted *p*-value less than 0.05 (Benjamini–Hochberg correction for multiple testing). PCA plots from significant DE genes were generated using base R libraries and ggplot2 (version 2.1.0).

Pathway analysis of significant gene sets was performed using the GAGE R/Bioconductor package^48^. Pathway activation/repression was determined by gene set enrichment analysis performed by GAGE with pathway annotations from Gene Ontology Consortium^49^, KEGG and Reactome^50^ databases. Significance was measured using an adjusted *p*-value less than 0.05 (Benjamini–Hochberg correction for multiple testing).

### Statistical analyses

Data were analyzed using Prism 6 for Mac OSX (Graphpad Inc.; La Jolla, CA). All dependent variables were analyzed separately using repeated measures ANVOAs, when appropriate Tukey HSD analysis was performed. Significance was accepted at the *α* < 0.05 level and data are reported as mean ± SE.
