# Correction: Multiscale analysis of a regenerative therapy for treatment of volumetric muscle loss injury

**DOI:** 10.1038/s41420-018-0080-3

**Published:** 2018-07-23

**Authors:** Carlos A. Aguilar, Sarah M. Greising, Alain Watts, Stephen M. Goldman, Chelsea Peragallo, Christina Zook, Jacqueline Larouche, Benjamin T. Corona

**Affiliations:** 1 Department of Biomedical Engineering, University of Michigan, Ann Arbor, MI USA; 2 Extremity Trauma and Regenerative Medicine, United States Army Institute of Surgical Research, Fort Sam Houston, San Antonio, TX USA; 3 Massachusetts Institute of Technology - Lincoln Laboratory, Lexington, MA USA

## Abstract

**Correction to:** *Cell Death and Discovery* (2018) **4**, 33; 10.1038/s41420-018-0027-8; published online 20 February 2018.

**Correction to:** *Cell Death and Discovery* (2018) **4**, 33; 10.1038/s41420-018-0027-8; published online 20 February 2018.

Since the publication of this article, the authors noticed that the accession numbers for the datasets were not included in the publication. Also, the title for reference 34 was incorrect and should read “Transcriptional and chromatin dynamics of muscle regeneration after severe trauma.”

The original html and pdf versions of the paper have been rectified accordingly.

